# Early diagnosis and prognostic potential of RAC3 in bladder tumor

**DOI:** 10.1007/s11255-023-03781-0

**Published:** 2023-09-20

**Authors:** Shuo Wang, Zhuo Wei, Hui Shu, Yandong Xu, Zheqi Fan, Songtao Shuang, Pei Li, Pan Lu, Chang Ye

**Affiliations:** 1 Postgraduate Training Base of Xiaogan Central Hospital, Jinzhou Medical University, Xiaogan, 432000 China; 2 Department of Urology, The Central Hospital of Xiaogan, Xiaogan, 432000 China; 3 Department of Pathology, The Central Hospital of Xiaogan, Xiaogan, 432000 China; 4 Department of Gastroenterology, The Central Hospital of Xiaogan, Xiaogan, 432000 China

**Keywords:** Bladder tumor, RAC3, Early diagnosis, Prognostic potential

## Abstract

**Background and purpose:**

Bladder tumors are among the most prevalent malignancies in the urinary system, and RAC3 has been linked to various types of cancer. This article seeks to explore the potential of RAC3 as both an early diagnostic marker for bladder tumors and a novel therapeutic target.

**Methods/patients:**

The expression of RAC3 in bladder tissue was detected using immunohistochemical staining. Additionally, the protein expression of RAC3 was measured and quantified through enzyme-linked immunosorbent assay (ELISA). Subsequently, the correlation between the expression level of RAC3 and bladder tumors was investigated through multifactorial analysis and survival analysis.

**Results:**

Our findings revealed that RAC3 expression was upregulated in bladder tumor tissues. Moreover, we observed higher levels of RAC3 expression in the serum and urine of patients with bladder tumors compared to those with non-bladder tumors. Additionally, we identified a significant positive correlation between RAC3 expression levels and the stage, degree of differentiation, and infiltration of bladder tumors. Importantly, high RAC3 expression emerged as an influential factor in the poor prognosis of bladder tumors, as patients with high RAC3 expression exhibited a lower overall survival rate than those with low RAC3 expression.

**Conclusion:**

Based on our results, RAC3 shows promise as both a marker for early diagnosis of bladder tumors and a potential therapeutic target.

## Introduction

Bladder tumors are a type of malignant tumor in the urogenital system with a high incidence and mortality rate. The prevalence of bladder tumors is increasing annually. Non-muscle-invasive bladder cancer (NMIBC), which is confined to the mucosa, accounts for approximately 75% of cases. The remaining 25–30% of patients have muscle-invasive bladder cancer (MIBC), where cancer has invaded deeper layers of the bladder wall or has formed metastases [1]. High-grade invasive bladder tumors have been associated with high recurrence and mortality rates compared to other types of bladder tumors. Despite achieving favorable short-term efficacy in many cases, the 5 years survival rate for bladder tumors remains low [2]. Currently, there are no biomarkers that significantly impact the prognosis of patients with bladder tumors. Therefore, the investigation of new biomarkers holds great significance for the treatment and prognosis of patients with bladder tumors.

RAC3, also known as ras-related C 3 botulinum substrate 3, belongs to the Rho family of small GTPases. Like other members of this family, RAC3 is essentially a molecular switch, being “on” in the GTP-bound state and “off” in the GDP-bound state [3, 4]. The RAC family has been implicated in various processes related to malignant tumorigenesis, including proliferation, apoptosis, autophagy, metastasis, invasion, and regulation of cellular signaling pathways [5, 6]. RAC3 is widely expressed in different tissues and is particularly prominent in the nervous system, with significant expression present in the developing brain and in projection neurons of the spinal cord and dorsal root ganglia [7]. Current studies have shown that Rac3 is involved in a variety of malignant tumors, including breast, endometrial, and lung cancers. It plays an important role in adhesion, cell migration, cell invasiveness, and apoptosis [8–10]. Additionally, Rac3 has been found to have a significant role in distinguishing prostate cancer epithelial cells from benign prostatic hyperplasia epithelial cells [11]. However, there is limited research on the relationship between Rac3 and bladder tumors, and further investigation is needed.

In this study, we conducted several analyses related to RAC3 in bladder tumors. First, we compared the expression of RAC3 between normal bladder tissues and bladder tumor tissues at different stages and grades. Subsequently, we examined the correlation between RAC3 expression and the prognosis of bladder tumor patients. Additionally, we compared the expression of RAC3 in the serum and urine of patients with bladder tumors and patients with chronic cystitis. Last, we investigated the potential of RAC3 as a biomarker for early diagnosis of bladder tumors.

### Methods/patients

#### Tissue samples, serum and urine samples

In this study, bladder tissues were collected from a total of 70 patients who underwent bladder surgery between 2018 and 2022. The patients’ ages ranged from 56 to 88 years, with a mean age of 62.3 years. The male-to-female ratio was 2.6:1. Based on the pathological diagnosis, the patients were divided into two groups. The bladder tumor group consisted of 60 patients. Their ages ranged from 61 to 88 years, with a mean age of 66.1 years. The male-to-female ratio in this group was 3:1. The remaining 10 patients belonged to the non-bladder tumor group. Their ages ranged from 56 to 71 years, with a mean age of 63.6 years. The male-to-female ratio in this group was 3:2. Among the 60 patients with tumors, 53 had TNM stage I-II, and 7 had stage III-IV. The pathological grading included 30 cases of high-grade and 30 cases of low-grade tumors. Based on the stage and grade of the tumors, the patients were further divided into subgroups. There were 15 patients each in the high-grade infiltrating group, high-grade non-infiltrating group, low-grade infiltrating group, and low-grade non-infiltrating group.

In addition, serum and morning urine samples were collected from 60 patients who visited our hospital for bladder disorders from 2022 to 2023. The patients’ ages ranged from 53 to 78 years, with a mean age of 62.3 years. The male-to-female ratio was 2.1:1. Based on the pathological diagnosis, the patients were divided into two groups. The bladder tumor group consisted of 36 patients. Their ages ranged from 55 to 78 years, with a mean age of 66.4 years. The male-to-female ratio in this group was 2.2:1. The remaining 14 patients belonged to the chronic cystitis group. Their ages ranged from 53 to 75 years, with a mean age of 61 years. The male-to-female ratio in this group was 1.8:1. Among the 36 patients with tumors, 25 had high-grade pathology and 11 had low-grade pathology. They were further divided into the high-grade group and the low-grade group.

The above pathology results were verified by pathological analysis. The collected samples were afterward stored in a refrigerator at – 80 °C. The pathologic tissue type of all patients with tumors included in this study was uroepithelial carcinoma with no other pathological subtypes, and none of the patients suffered from other malignancies or serious underlying medical conditions. All materials used in this study were obtained with the consent of all patients and approved by the organization’s ethics committee.

#### Immunohistochemistry

The expression of RAC3 was analyzed in bladder tumor tissues, adjacent non-tumor tissues, and normal bladder tissues using immunohistochemical staining. The immunohistochemical staining procedure involved the following steps: sectioning, de-waxing, antigen retrieval, blocking, incubation with a primary antibody (Anti-RAC3 antibody, ab124943; dilution: 1:70), incubation with a secondary antibody, staining with 3,3′-diaminobenzidine (DAB), counterstaining with hematoxylin, decolorization with hydrochloric acid and ethanol, blueing, dehydration, and coverslipping.

The scoring criteria for staining intensity on slides in this study are as follows: no staining is scored as 0, light brown is scored as 1, brown is scored as 2, and dark brown is scored as 3. The criteria for the proportion of cells with positive staining are as follows: 0 is scored as ≤ 5%, 1 is scored as > 5–25%, 2 is scored as > 25–50%, and 3 is scored as > 50%. To obtain the final score, the two scores (intensity and proportion) are multiplied. The final expression is then categorized as follows: scores of 0–3 are considered negative expression, scores of 4–6 are considered weak positive expression, and scores of 7–9 are considered strong positive expression [9]. The final scoring results are evaluated by two independent pathologists who are blinded to the clinical data.

#### ELISA

In this study, the expression of RAC3 in serum and urine samples from patients with chronic cystitis and patients with different stages of bladder tumors was determined using the RAC3 ELISA kit. The RAC3 ELISA kit utilizes a double antibody one-step sandwich method. The experimental steps involved in the assay are as follows: coating plate, sealing, washing, adding sample, coating, incubation, washing, incubation with antibody, washing, incubation with enzyme complex, washing, adding colorimetric substrate, stopping the reaction, and measuring the results. Results judgment: with the standard concentration as the abscissa and the corresponding OD value as the ordinate, the linear regression curve of the standard is drawn, and the concentration of each sample is calculated according to the curve equation.

### Statistical analysis

In this study, statistical analysis was conducted using SPSS 26.0 Software (SPSS, Chicago, IL, USA), and figures were generated using GraphPad Prism 9.5.1 Software. The chi-square test was employed to compare measurement data, while the *t* test was used for count data comparisons. Univariate and multivariate survival analyses were performed using Cox regression models. Kaplan–Meier survival analysis was utilized to assess overall patient survival. A significance level of *P* < 0.05 was considered statistically significant. All P values reported are from two-tailed tests.

## Result

### RAC3 is an over-expressed gene in urinary bladder tumors

**Fig. 1:**
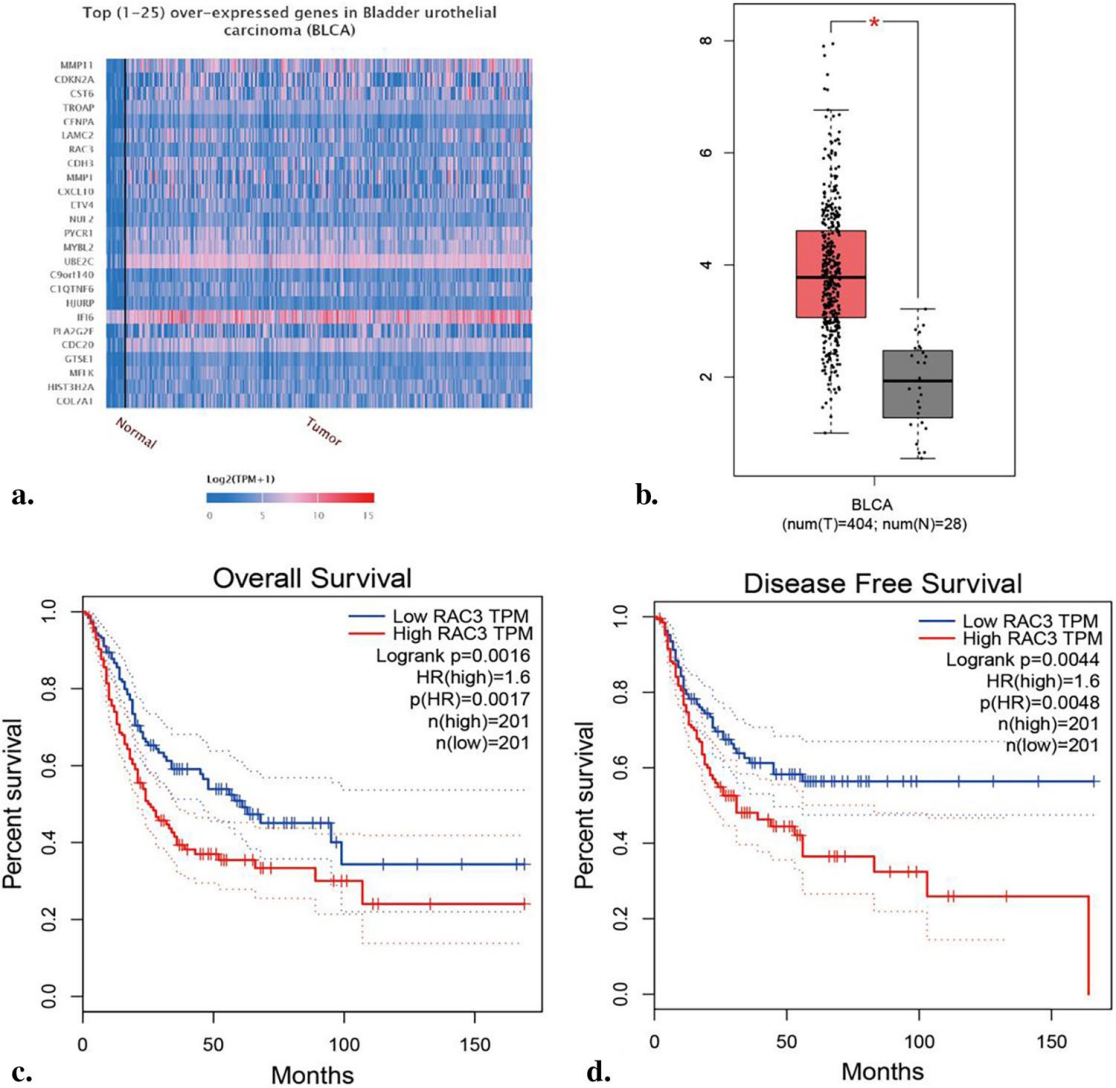
RAC3 overexpression in bladder tumor tissues: **a** The UALCAN database (http://ualcan.path.uab.edu/index.html) was utilized to analyze the expression levels of the top 25 genes that are overexpressed in bladder cancer. **b** The GEPIA database (http://gepia.cancer-pku.cn/) was used to analyze the transcript levels of RAC3 in both bladder cancer tissue (T) and normal bladder tissue (N). **c** Bioinformatics analysis conducted using the GEPIA database revealed a correlation between the transcript levels of RAC3 and the overall survival of bladder cancer patients. **d** Additionally, bioinformatics analysis utilizing the GEPIA database demonstrated the relationship between RAC3 transcript levels and disease-free survival among bladder cancer patients

The UALCAN database contains graded TCGA 3 RNA-seq and clinical data for 31 cancer types, allowing the identification of highly and lowly expressed genes within each specific cancer type [12]. GEPIA, on the other hand, is a tool used for cancer and normal gene expression profiling and interactive analysis. It offers various features such as differential expression analysis, profile mapping, correlation analysis, patient survival analysis, similar gene detection, and dimensionality reduction analysis [13]. In our study, we utilized the UALCAN database to perform bioinformatics analysis, which revealed that RAC3 is among the genes that are overexpressed in bladder tumors. To further validate this finding, we conducted an additional analysis using the GEPIA database and confirmed that RAC3 expression is upregulated in bladder tumor tissues compared to normal bladder tissue. Moreover, our analysis demonstrated that patients with higher RAC3 expression exhibited shorter overall survival and disease-free survival rates when compared to those with lower RAC3 expression (Fig. 1).

### RAC3 is highly expressed in high-grade invasive bladder tumor tissues

**Fig. 2:**
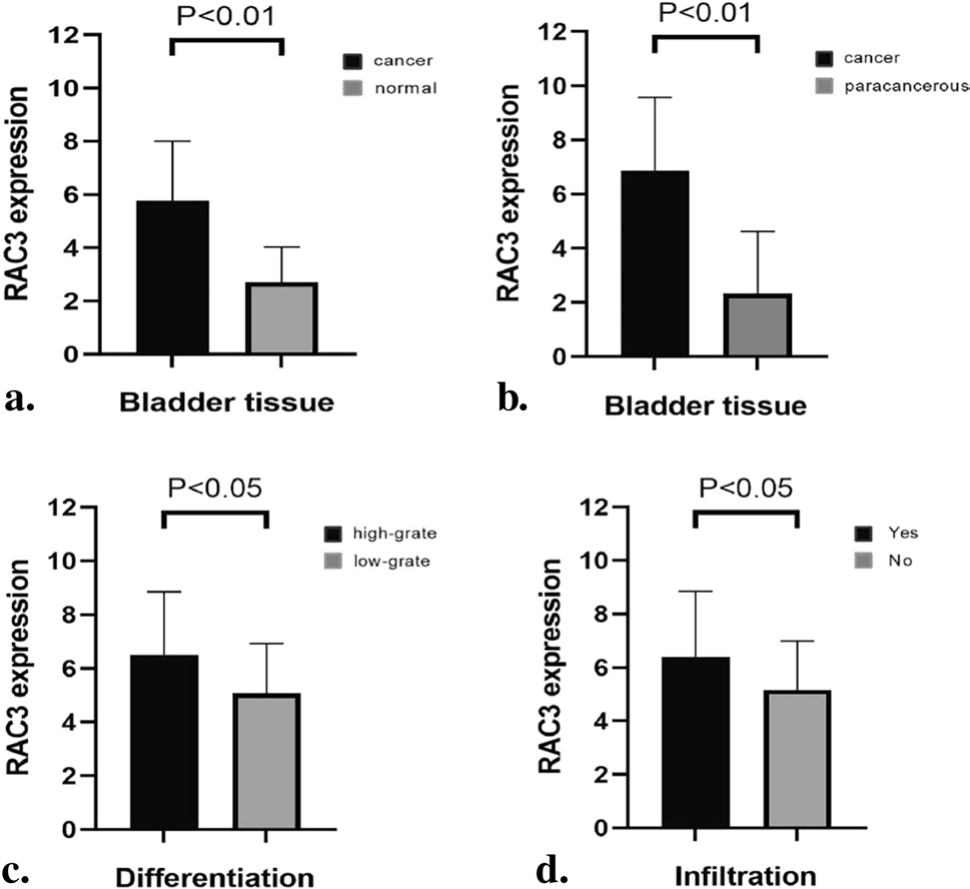
High expression of RAC3 in bladder tumor tissues: **a** RAC3 expression in bladder tumor tissues and normal bladder tissues adjacent to the cancer in 15 patients. **b** RAC3 expression in bladder tissues of 60 bladder tumor patients and 10 non-bladder tumor patients. **c** RAC3 expression in bladder tumor tissues of 60 patients with different grades of differentiation. **d** RAC3 expression in bladder tissues of 60 bladder tumor patients with different depths of infiltration

**Fig. 3:**
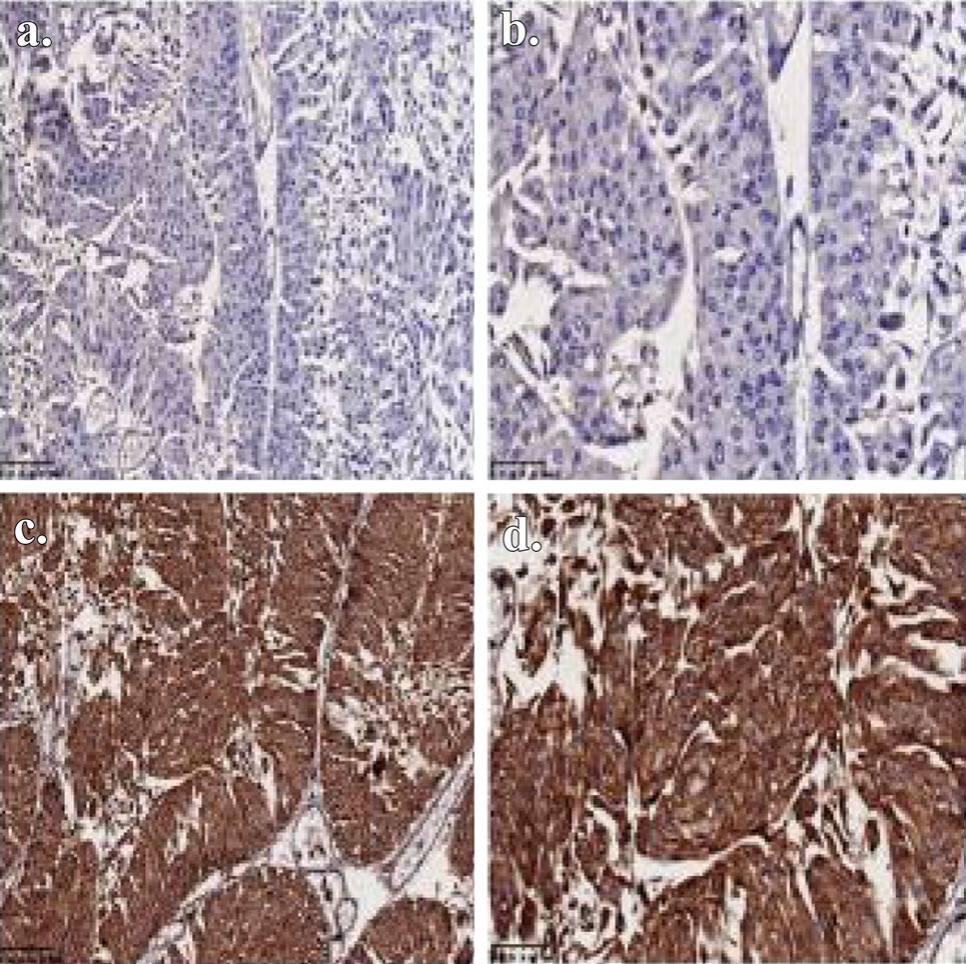
Immunohistochemical staining of RAC3 expression in different tissues: non-tumor bladder tissues(–): **a** × 200 and **b** × 400; Bladder tumor tissues (+ +  + +): **c** × 200 and **d** × 400

To validate our findings, we examined the expression of RAC3 in bladder tissues from 60 patients with bladder tumors and 10 patients without bladder tumors. We observed that RAC3 expression was significantly higher in bladder tumor tissues compared to normal bladder tissues in individuals without bladder tumors (Fig. 2a). The expression of RAC3 in different tissues is shown in Fig. 3. Additionally, in a subset of 15 randomly selected patients with bladder tumors, we found high RAC3 expression in bladder tumor tissues compared to adjacent normal bladder tissues (taken as tissues 2 cm from the edge of the tumor) (Fig. 2b). Furthermore, we conducted an analysis to explore the differences in RAC3 expression in 30 bladder tumor tissues with varying grades and levels of tumor infiltration. The results revealed a positive correlation between RAC3 expression and bladder tumor grade and infiltration depth (Fig. 2c, d).

### RAC3 was highly expressed in the serum and urine of patients with bladder tumors

**Fig. 4:**
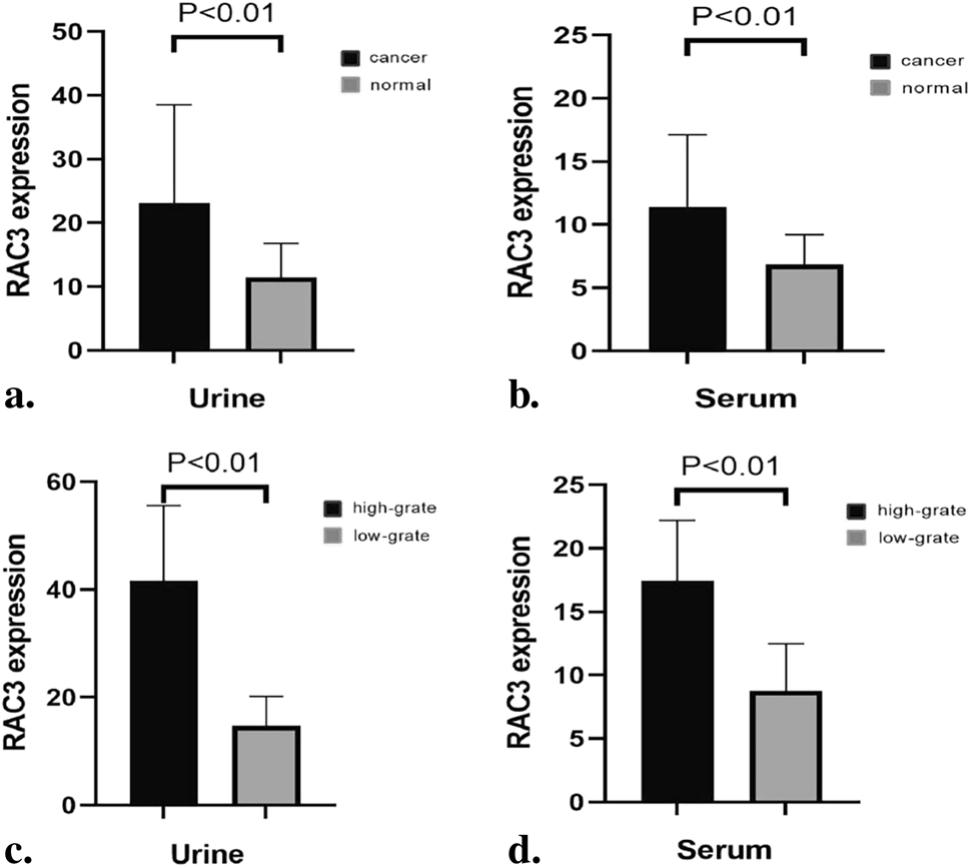
RAC3 is highly expressed in serum and urine of patients with bladder tumors (*n* = 50): **a** RAC3 expression in urine of 36 patients with bladder tumors and 14 patients with chronic cystitis. **b** RAC3 expression in serum of 36 patients with bladder tumors and 14 patients with chronic cystitis. **c** RAC3 expression in urine of 36 patients with bladder tumors with different grades of differentiation. **d** RAC3 expression in serum of 36 patients with bladder tumors with different grades of differentiation

Based on a previous study, we conducted further analysis on 36 patients with bladder tumors and 14 patients with chronic cystitis. Our results revealed a significant increase in the expression of RAC3 in serum and urine samples from patients with bladder tumors compared to those with chronic cystitis. Additionally, patients with high-grade tumors exhibited higher levels of RAC3 expression in serum and urine compared to patients with low-grade tumors (Fig. 4).

### RAC3 expression is associated with the clinical regression of bladder tumors

**Fig. 5:**
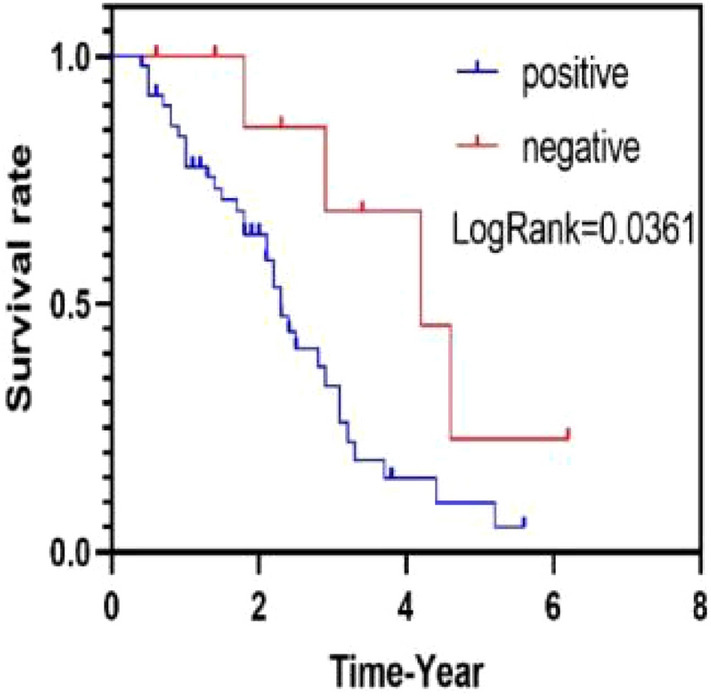
Kaplan–Meier analysis of overall survival of 60 patients with bladder tumor carcinoma by RAC3 expression. Patients with negative expression of RAC3 had significantly longer survival time compared to the positive expression group (Log-Rank test, *P* < 0.05)

A more comprehensive analysis of the data related to Result 2.2 revealed the following results. The correlation between RAC3 expression and various clinicopathological characteristics, including gender, age, smoking history, TNM stage, degree of differentiation, and depth of infiltration, was examined in the 60 patients with bladder tumors (Table 1). The expression of RAC3 was found to be correlated with smoking history, TNM stage, depth of infiltration, and degree of differentiation. However, no significant correlation was observed between RAC3 expression and age or gender. To evaluate the impact of RAC3 expression on overall survival in patients with bladder tumors, univariate and multivariate analyses were conducted using Cox regression models. The univariate analysis demonstrated that RAC3 expression, tumor TNM stage, depth of infiltration, and degree of differentiation influenced the prognosis of bladder tumors. In the multivariate analysis, RAC3 expression, TNM stage, depth of infiltration, and degree of differentiation were identified as independent risk factors for bladder tumor prognosis (Table 2). Furthermore, Kaplan–Meier analysis revealed that patients with RAC3-positive bladder tumors had a lower overall survival rate compared to those with negative RAC3 expression (Fig. 5).

**Table 1:** Demographic data and correlation between RAC3 expression and clinicopathological features in 60 patients with bladder tumors

	RAC3 expression			Total	Chi-square value	*P* value
	Negative	Weakly positive	Strongly positive			
Total cases	9	37	14	60		
Smoking						
Yes	2	33	13	38	22.177	0.000**
No	7	4	1	12		
Age						
> 65	5	24	13	42	3.069	0.216
< 65	4	12	2	18		
Gender						
Man	7	25	13	45	3.508	0.173
Woman	2	12	1	15		
TNM state						
I–II	6	11	39	53	17.56	0.000**
III–IV	3	0	4	7		
Infiltration						
Yes	5	12	13	30	14.964	0.001**
No	4	25	1	30		
Differentiation						
High-grade	5	14	11	30	6.872	0.032*
Low-grade	4	23	3	30		

*Indicates statistical significance (*P < 0.05, **P < 0.01)

**Table 2:** Univariate and multifactorial analysis of factors related to overall survival in 60 patients with bladder tumors

Variables	Univariate analysis			Multivariate analysis		
	HR	95% CI	*P* value	HR	95% CI	*P* value
RAC3expression	1.252	1.059–1.480	0.009**	1.238	1.036–1.480	0.019*
TNM state	3.701	1.359–10.080	0.010*	4.259	1.406–12.912	0.010*
Infiltration	2.454	1.109–5.427	0.027*	4.114	1.132–5.133	0.022*
Differentiation	3.075	1.543–6.131	0.001**	2.444	1.112–5.371	0.026*
Gender	0.974	0.453–2.094	0.946			
Age	1.052	0.530–2.088	0.886			
Smoking	0.758	0.294–1.954	0.567			

*Indicates statistical significance (*P < 0.05, **P < 0.01)

## Discuss

The pathogenesis of bladder tumors is a complex process that includes multiple factors, genes, and steps involved in tumor formation. A range of treatment options are available for bladder tumors, which may vary depending on the stage and grade of the tumor. In recent years, there have been advancements in research that have led to the discovery of new treatments, alongside standard treatment options, such as tri modality combination therapy (TMT) and nanotechnology. However, despite the availability of these treatment options, high recurrence rates continue to be a significant challenge in the treatment of bladder cancer [1, 14, 15]. Therefore, early diagnosis and identification of new therapeutic targets are critical for improving the outcome and prognosis of patients with bladder tumors.

Rac3 is highly expressed in many malignant tumors and is involved in the regulation of tumorigenesis through different mechanisms [16–19]. Rubio et al. [20] found that RAC3 was expressed at a high level in human colon tumors and showed a significant increase in tumor growth rate and colon cancer incidence by inoculating cells overexpressing RAC3 into nude mice. It was also shown that RAC3 promotes tumor cell resistance to chemotherapeutic drugs by inhibiting apoptosis and autophagy. In addition, Rosenberg et al. [8, 21] found that RAC3 promotes invasion and metastasis in breast and lung adenocarcinomas, and inhibition of RAC3 expression in tumor cells significantly reduced the invasion and metastasis of lung adenocarcinoma cells. However, at present, there are few studies on the expression of RAC3 in bladder tumors, and most of them remain at the stage of bioinformatics analysis. For example, Cheng et al.’s study based on the TCGA database showed that RAC3 was upregulated in patients with bladder tumors, and that RAC3 may promote the proliferation, migration, and invasion of bladder tumor cells through the PYCR1/JAK/STAT signaling pathway [2, 22], but it has not yet been demonstrated at the clinical level. Therefore, it is particularly important to explore the clinical significance of RAC3 in patients with bladder tumors.

In our study, we first derived that RAC3 was associated with bladder tumors by searching and analyzing the TCGA database. We then detected the expression of RAC3 in bladder tumor tissues from patients with bladder tumors, normal tissues adjacent to cancer, and non-bladder tumor tissues in different individuals by immunohistochemical staining. The results showed that RAC3 was highly expressed in bladder tumor tissues and more significantly up-regulated in high-grade, invasive bladder tumor tissues. Second, by characterizing the expression of RAC3 with clinical case characteristics of bladder tumor patients, we found that the expression of RAC3 in patients was associated with smoking history, TNM stage, depth of infiltration, and degree of differentiation, and there was no significant correlation with age or gender, and that the expression of RAC3, TNM stage, depth of infiltration, and degree of differentiation were independent risk factors for the prognosis of bladder tumors. Subsequently, survival analysis by follow-up of bladder tumor patients revealed that bladder tumor patients with upregulated RAC3 expression had reduced overall survival. All of the above indicated that RAC3 expression was associated with bladder tumor grade, depth of infiltration, treatment, and prognosis, which is the same as the results demonstrated by the TCGA database and Cheng et al. [2]. Therefore, it is reasonable to believe that RAC3 has the promise to be a new therapeutic target for bladder tumors and even improve patient prognosis.

The importance of early detection, diagnosis, and treatment of any disease has always been recognized. As the study progressed, we further examined the expression of RAC3 in serum and urine samples from patients with chronic cystitis and bladder tumors. The results showed that patients with bladder tumors had elevated levels of RAC3 expression in serum and urine compared to patients with chronic cystitis. In addition, the expression of RAC3 was more significantly upregulated in the serum and urine of patients with highly graded bladder tumors. This is the first report that RAC3 is highly expressed in serum and urine in addition to tumor tissue in patients with bladder tumors. Therefore, we believe that RAC3 is expected to be a potential tumor marker for early diagnosis of bladder tumors.

Constrained by the fact that bladder cancer itself has few or no early symptoms, leading to late staging and delayed treatment for most bladder cancer patients when it is detected, the prognosis of bladder cancer is poor, despite the existence of a wide range of therapeutic options. According to the results of this study, RAC3 has greater potential for early diagnosis of bladder cancer and improving patient prognosis. Testing the expression of RAC3 in serum and urine of high-risk individuals with bladder disease symptoms or with a family history of the disease, and performing more detailed examinations if abnormal results are found, could help in the early detection of bladder cancer patients. In other words, this method can be used as an initial screening tool for bladder cancer. In terms of prognosis, RAC3 can be considered as a new therapeutic target, leading to the research of novel targeted drugs, which is of great significance for bladder cancer patients with upregulated RAC3 expression.

Although the results of this study demonstrate the importance of RAC3 in bladder tumors, certain limitations remain. First, the relatively small number of cases included in the analysis and the fact that the patients predominantly had stage I and II bladder tumors created some limitations in this study. In future studies, we will increase the sample size and extend the study period to minimize the impact caused by these limitations. In addition, the focus of this study was to investigate this clinical phenomenon and did not address the specific mechanism of this relationship between RAC3 and bladder cancer. To address this issue, we will explore the mechanism of their relationship in future studies. We also hope and recommend that more interested scholars participate in this study, as it is of great significance to the diagnosis and treatment of bladder cancer patients, and even to the entire medical community.

## Conclusion

In conclusion, in this study, we found that RAC3 promotes the proliferation, differentiation, and invasion of bladder tumors, and its presence is associated with poor prognosis. This finding provides a new idea for the early diagnosis of bladder tumors and introduces a new therapeutic target for the treatment of bladder tumors. However, more experimental studies are still needed to determine whether it can eventually be applied to the clinic.

## Data Availability

The data involved in this article are available, but due to the privacy of the patients involved, and the large amount of data, it is not convenient to fully open, if the reader wants, you can apply to the corresponding author of this article, if the application is successful, you can get the data used in this article.
